# Retraction: Identification and characterization of genes related to salt stress tolerance within segregation distortion regions of genetic map in F_2_ population of upland cotton

**DOI:** 10.1371/journal.pone.0275940

**Published:** 2022-10-21

**Authors:** The PLOS ONE Editors

The *PLOS ONE* Editors retract this article [1] because it was identified as one of a series of submissions for which we have concerns about authorship, competing interests, and peer review. We regret that the issues were not addressed prior to the article’s publication.

MShehzad, AD, AM, MShaban, MN, and FL did not agree with the retraction. XC, ZZ, and MK responded but expressed neither agreement nor disagreement with the editorial decision. YX, AK, MJA, and KW either did not respond directly or could not be reached.
